# One newly-recorded rare genus of Braconini (Hymenoptera, Braconidae, Braconinae) from Hainan, China

**DOI:** 10.3897/BDJ.14.e210019

**Published:** 2026-08-26

**Authors:** Ming-Min Zheng, Yang Li, Cornelis van Achterberg, Jia-Chen Zhu, Xue-xin Chen

**Affiliations:** 1 Sichuan Provincial Forestry and Grassland Key Laboratory of Biodiversity Conservation and Sustainable Community Development in Giant Panda National Park, College of Chemistry and Life Sciences, Chengdu Normal University, Chengdu, China Sichuan Provincial Forestry and Grassland Key Laboratory of Biodiversity Conservation and Sustainable Community Development in Giant Panda National Park, College of Chemistry and Life Sciences, Chengdu Normal University Chengdu China; 2 State Key Laboratory of Rice Biology, Ministry of Agriculture and Rural Affairs Key Lab of Molecular Biology of Crop Pathogens and Insects, Zhejiang Key Laboratory of Biology and Ecological Regulation of Crop Pathogens and Insects, and Institute of Insect Sciences, Zhejiang University, Hangzhou, China State Key Laboratory of Rice Biology, Ministry of Agriculture and Rural Affairs Key Lab of Molecular Biology of Crop Pathogens and Insects, Zhejiang Key Laboratory of Biology and Ecological Regulation of Crop Pathogens and Insects, and Institute of Insect Sciences, Zhejiang University Hangzhou China; 3 College of Advanced Agricultural Sciences, Zhejiang A&F University, Hangzhou, China College of Advanced Agricultural Sciences, Zhejiang A&F University Hangzhou China

**Keywords:** *
Hemiglyptinus
*, new record, Oriental

## Abstract

**Background:**

*Hemiglyptinus* Shenefelt, 1978 is a monotypic genus of the subfamily Braconinae (Hymenoptera, Braconidae) with only one described species worldwide. The biology of this genus is still unknown. Up to present, *Hemiglyptinus* has not been recorded from China.

**New information:**

This study reports the first record of the genus *Hemiglyptinus* in the Hainan Province of China; the newly-recorded species, *Hemiglyptinus flavus* (Ashmead, 1905), is re-described and illustrated.

## Introduction

The rare genus *Hemiglyptinus* Shenefelt, 1978 (new name for the junior homonym *Hemiglyptus* Ashmead, 1905; tribe Braconini) occurs only in the Oriental Region and so far only known from the Philippines (Luzon) (Yu et al. 2016, Quicke et al. 2023). Amongst the Braconinae in the collection of the College of Chemistry and Life Sciences of Chengdu Normal University (Chengdu), *Hemiglyptinus flavus* (Ashmead, 1905) was discovered for the first time since its description amongst specimens from Hainan Province (southern China). This identification was confirmed after examination of the holotype, including its original description and photographs (available on the USNM website). In the present paper, the diagnosis of the genus *Hemiglyptinus* is reviewed and the newly-recorded species is re-described and illustrated for the first time.

## Materials and methods

The specimen of **Hemiglyptinus* flavus* (Ashmead, 1905) from Hainan was collected with a Malaise trap and kept in 100% ethanol before being point mounted on a small triangular card. The specimen is deposited in the College of Chemistry and Life Sciences, Chengdu Normal University, Chengdu (**CDNU**).

The terminology and measurements used in this paper, follow van Achterberg (1988) and van Achterberg (1993). The medial length of the third metasomal tergite is measured from the posterior border of the second suture to the posterior margin of the tergite; **POL** = postocellar line; **OOL** = ocular-ocellar line.

Photographs were taken with a Canon 6D mark II digital camera with Laowa 25 mm f2.8 + 2.5-5.0x lens and claws, apex of antenna and ovipositor with Mitutoyo 10x lens. The photos were slightly processed (mainly cropped and the background modified) in Photoshop 2026. For the descriptions and measurements, an Olympus SZX7 stereomicroscope was used.

## Taxon treatments

### Hemiglyptinus

Shenefelt, 1978

232CC8EB-6661-52BD-BC64-15658692C3DC

Hemiglyptus Ashmead (1905): 412.
Hemiglyptinus Shenefelt (1978): 1687. Replacement name for *Hemiglyptus* Ashmead, 1905 not Horn, 1889.
Hermiglyptus flavus Ashmead, 1905Ashmead (1905): 412. by Monotypy

#### Diagnosis

**Figure 1.**
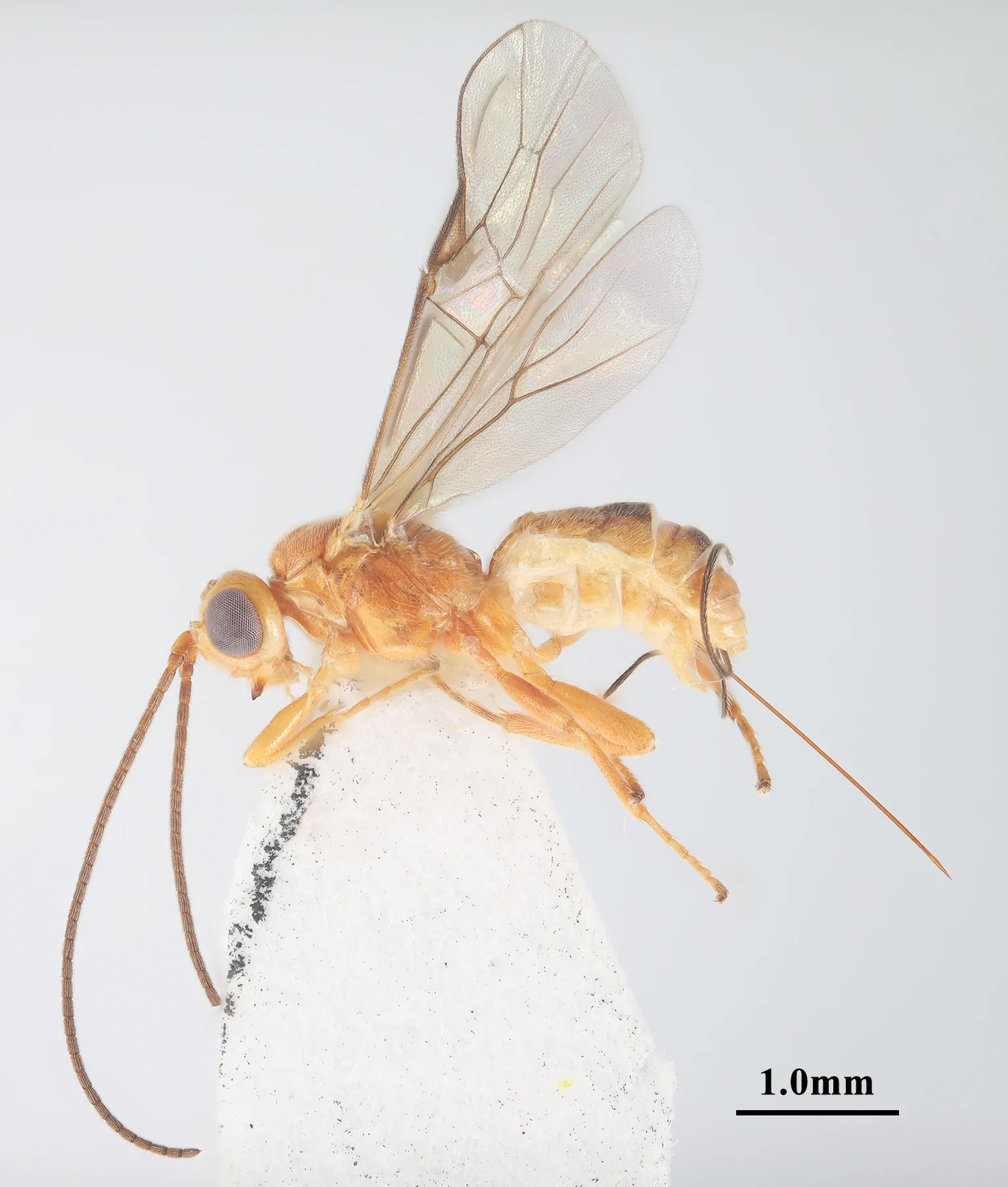
**Hemiglyptinus* flavus* (Ashmead, 1905), ♀, habitus, lateral aspect.

**Figure 2.**
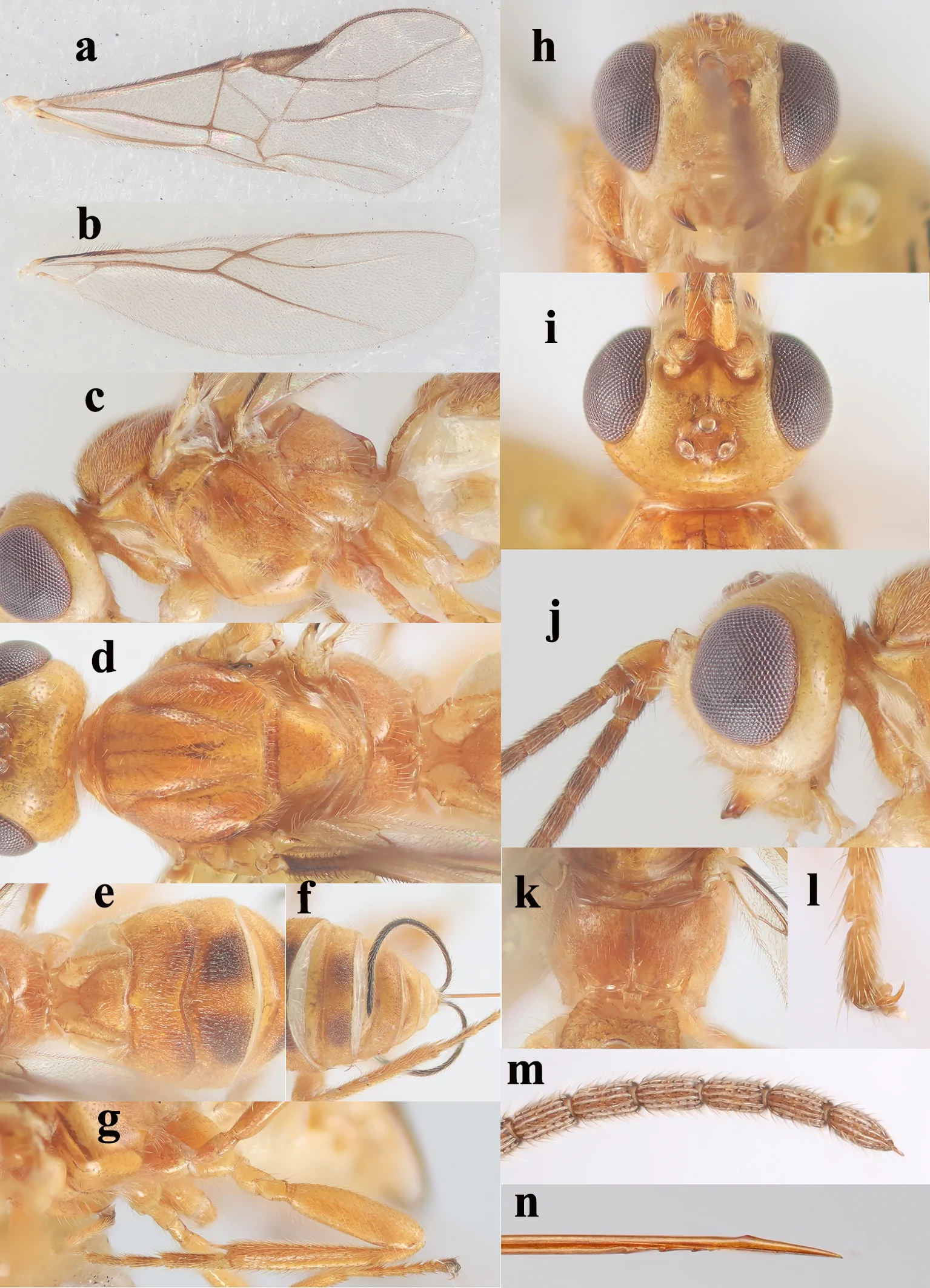
**Hemiglyptinus* flavus* (Ashmead, 1905), ♀. **a** fore-wing; **b** hind-wing; **c** mesosoma, lateral aspect; **d** mesosoma, dorsal aspect; **e** first to third metasomal tergites, dorsal aspect; **f** fourth to sixth metasomal tergites, dorsal aspect; **g** hind leg, lateral aspect; **h** head, anterior aspect; **i** head, dorsal aspect; **j** head and scapus outer side, lateral aspect; **k** metanotum and propodeum, dorsal aspect; **l** apex of hind tarsus, lateral aspect; **m** apex of antenna; **n** apex of ovipositor, lateral aspect.

**Figure 3.**
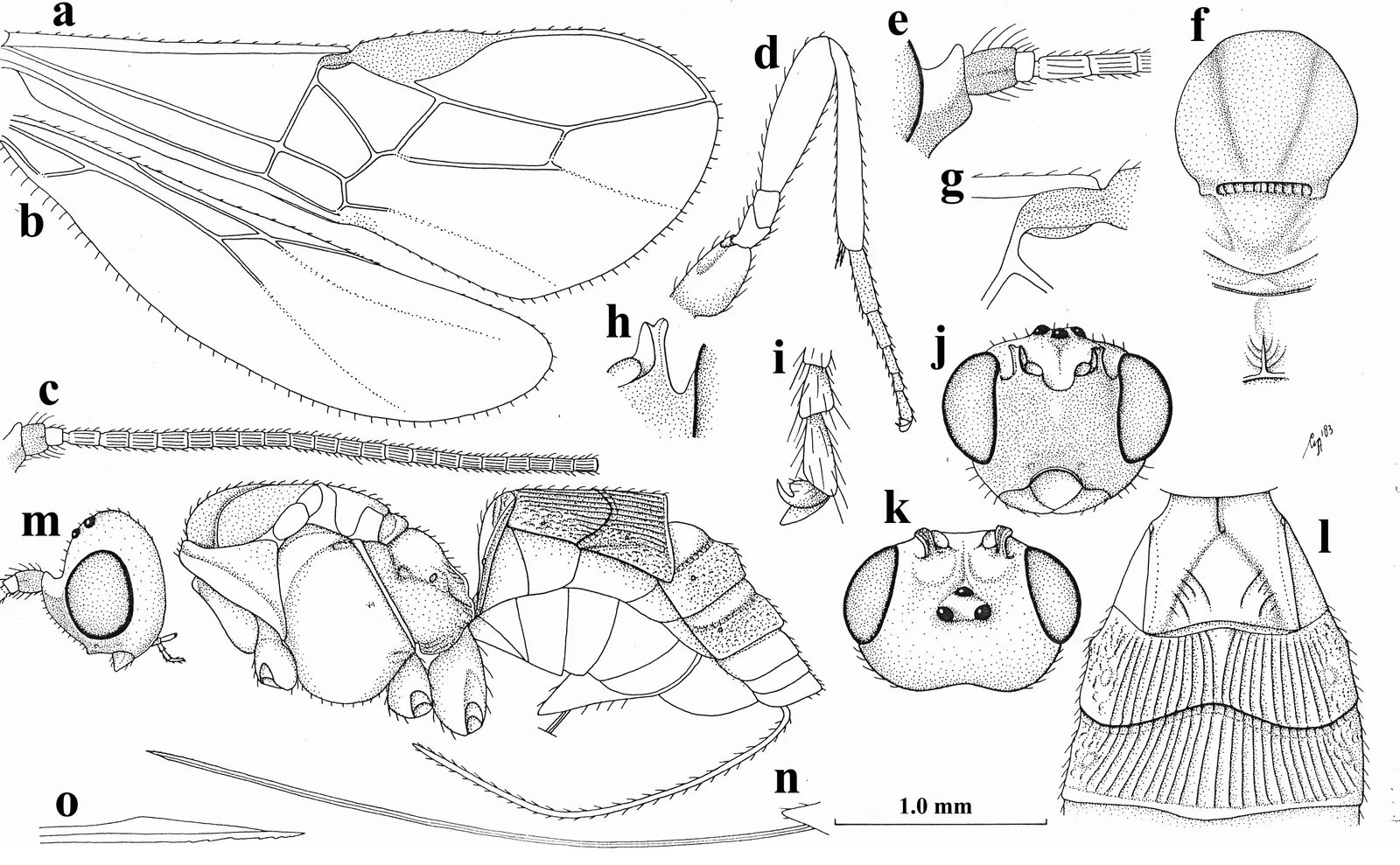
*Hemiglyptinus flavus* (Ashmead, 1905), ♀, holotype. **a** fore-wing; **b** hind-wing; **c** antenna (except for apex); **d** hind leg, lateral aspect; **e** scapus outer side, lateral aspect; **f** mesosoma, dorsal aspect; **g** fore-wing vein 1-SR; **h** protruding outer margin of antennal socket, anterior aspect; **i** apex of hind tarsus, lateral aspect; **j** head, anterior aspect; **k** head, dorsal aspect; **l** first to third metasomal tergites, dorsal aspect; **m** habitus, lateral aspect; **n** ovipositor, lateral aspect; **o** apex of ovipositor, lateral aspect.

Body 3.2-3.8 mm. Outer margin of antennal socket strongly protruding (Fig. 2j, Fig. 3h and j); middle flagellar segments of antenna longer than wide (Fig. 1 and Fig. 3c); apical antennal segment rather acute, with short spine (Fig. 2m); in lateral view, scapus truncate and dorsally nearly as long as ventrally (Fig. 2j and Fig. 3e); antennal sockets strongly toothed antero-laterally (Fig. 2j and Fig. 3e); eye glabrous, weakly emarginated (Fig. 2h and Fig. 3j); face punctate (Fig. 2h and Fig. 3j); clypeus with dorsal carina (Fig. 2j); malar suture relatively short and indistinct (Fig. 2j and Fig. 3j); labio-maxillary complex normal, not elongate (Fig. 2j); frons sparsely punctate, slightly concave behind antennal sockets, with median groove (Fig. 2i and Fig. 3k); mesosoma largely smooth, except for some punctures (Fig. 2c and d); notauli developed and complete (Fig. 2d and Fig. 3f); mesoscutum punctate; scutellar sulcus comparatively wide, with developed crenulae (Fig. 2d and Fig. 3f); metanotum with weak and short mid-longitudinal carina anteriorly (Fig. 2k); propodeum largely smooth, except for a medio-longitudinal carina and lateral carinae posteriorly (Fig. 2k and Fig. 3f); angle between veins 1-SR and C+SC+R of fore-wing about 75°; fore-wing vein SR1 not reaching the tip (Fig. 2a and Fig. 3a); fore-wing vein 1-SR+M straight (Fig. 2a and Fig. 3a); fore-wing veins 1-SR and 1-M, veins r and 3-SR forming a straight line, respectively (Fig. 2a and Fig. 3a); fore-wing vein cu-a interstitial; fore-wing vein CU1b rather short (Fig. 2a and Fig. 3a); vein 3-CU1 of fore-wing slender; second submarginal cell of fore-wing relatively long, slightly narrowing distally (Fig. 2a and Fig. 3a); hind-wing vein SC+R1 distinctly longer than vein 1r-m (Fig. 2b and Fig. 3b); claws medium-sized, ventral lobe square (Fig. 2l and Fig. 3i); metasomal tergites (at least second and third) largely and coarsely striae-sculptured (Fig. 2e, f, Fig. 3l and m); first metasomal tergite without dorsal carinae, median area not convex, lateral grooves wide and crenulate (Fig. 2e); second and third metasomal tergites rather transverse (Fig. 2e and Fig. 3l); second to fifth metasomal tergites with antero-lateral areas (Fig. 1); hypopygium medium-sized and slightly apically acute, not emarginate medio-apically; ovipositor normal, subapically upper valve with nodus and its lower valve with teeth ventrally (Fig. 2n and Fig. 3o).

#### Distribution

Oriental (*China, Philippines).

#### Notes

This genus is new to the fauna of China and recorded first time outside Philippines.

### Hemiglyptinus flavus

(Ashmead, 1905)

08417F10-75AC-50BA-AD38-181D7FCF6581

#### Materials

- **Type status:** Other material. **Occurrence:** catalogNumber: 2026022; recordNumber: HN10, LSX1324; recordedBy: Shupeng Dong; individualCount: 1; sex: female; lifeStage: adult; occurrenceID: DE444153-7CE8-56B0-9005-6FEDC747DDED; **Taxon:** scientificName: **Hemiglyptinus* flavus* (Ashmead, 1905); **Location:** country: China; stateProvince: Hainan; locality: Nankai Town, rubber forest; verbatimLatitude: 19°4’43.32’’N; verbatimLongitude: 109°23’38.74’’E; **Identification:** identifiedBy: Yang Li; Cornelis van Achterberg; **Event:** samplingProtocol: Malaise trap; eventDate: 15.V-30.VI.2021; **Record Level:** institutionCode: Chengdu Normal University (**CDNU**)

#### Description

♀, length of body 3.8 mm, of fore-wing 3.5 mm, of ovipositor sheath 1.9 mm.

**Head**. Antenna with 32 segments; apical antennal segment rather acute, with short spine, 2.9 times longer than its maximum width (Fig. 2m); penultimate segment 2.1 times longer than its width and 0.7 times as long as apical antennomere; median segments 1.7 times longer than wide (Fig. 1); scapus 1.5 times longer than wide and truncate apically, dorsally nearly as long as ventrally in lateral view (Fig. 2j); third and fourth segments both 2.0 times their maximum width; length of maxillary palp 0.6 times longer than height of head; inner side of eye weakly emarginated (Fig. 2h); face punctate (Fig. 2h); clypeus flat and smooth; hypoclypeal depression 0.6 times as wide as minimum width of face (Fig. 2h); frons slightly concave behind antennal sockets, punctate, with a median groove (Fig. 2i); vertex punctate (Fig. 2i); OOL:diameter of posterior ocellus:POL = 10:4:5; in dorsal view length of eye 2.3 times temple; temples directly narrowed behind eyes, largely smooth, except for few punctures and long setae (Fig. 2i and j); malar suture indistinct; length of malar space 0.8 times basal width of mandible; mandible twisted, both teeth robust and subequal.

**Mesosoma**. Length of mesosoma 1.4 times its height (Fig. 2c); mesopleuron largely smooth, except for a few punctures; mesoscutum with dense setae; notauli complete, rather developed and with a few striae posteriorly (Fig. 2d); scutellar sulcus comparatively wide and with distinct crenulae; scutellum with dense setae; metanotum smooth and convex medially, with median carina (Fig. 2k); propodeum largely smooth, except for a medio-longitudinal carina and lateral carinae posteriorly (Fig. 2k).

**Wings**. Fore-wing (Fig. 2a): m-cu 0.5 times as long as 1-M; 1-SR+M straight and 1.3 times as long as 1-M; r:3-SR:SR1 = 9:30:44; 2-SR:3-SR:r-m = 9:15:5; r-m largely not sclerotised; CU1b 0.3 times as long as 3-CU1. Hind-wing (Fig. 2b): with 3 hamuli on R1; 1r-m more or less straight; SC+R1:2-SC+R:1r-m = 25:5:9.

**Legs**. Tarsal claws with long bristly setae ventrally (Fig. 2l); length of femur, tibia and basitarsus of hind leg 3.6, 7.6 and 4.9 times their maximum width, respectively; hind tibial spurs both 0.4 times as long as hind basitarsus; hind tibia with a distinct subapical row of long, thick setae; hind femur, tibia and tarsus densely setose, setae of tarsus short (Fig. 2g).

**Metasoma**. Length of first metasomal tergite about as long as its apical width; lateral area of first tergite comparatively wide, lateral grooves sparsely crenulate (Fig. 2e); second tergite 3.5 times wider than medially long, largely coarsely striate-sculptured, except for medio-anteriorly (Fig. 2e); second suture wide, strongly sinuate and crenulate (Fig. 2e); third tergite largely longitudinally striate-sculptured (Fig. 2e); fourth and fifth tergites rugose, the following tergites smooth and shiny (Fig. 2f); hypopygium acute apically, reaching beyond the level of apex of metasoma; ovipositor sheath 0.54 times as long as fore-wing.

**Colour**. Pale brownish-yellow (Fig. 1); second to fourth metasomal tergites with pair of dark brown marks sublaterally (second tergite only postero-sublaterally) (Fig. 2e and f); antenna (except scapus) brown (Fig. 1); hind tarsus brownish (Fig. 2g); mandible apically, claws and ovipositor sheath dark brown; wing membrane hyaline, pterostigma and veins brown (Fig. 2a and b).

#### Distribution

Oriental (*China- Hainan, Philippines).

#### Notes

When compared with the holotype of *Hemiglyptinus flavus* (Ashmead, 1905) from the Philippines, the Hainan specimen exhibits some differences in metasomal colouration: second to fourth metasomal tergites with pair of dark brown marks sublaterally, while second tergite only postero-sublaterally, whereas in the type specimen, the metasoma is uniformly brownish-yellow. Apart from these colour differences, however, no structural differences were observed between the two specimens. In the Braconinae, colour patterns of the metasoma can be highly variable and such differences are often correlated with environmental factors, including temperature. Therefore, these colour differences alone are not sufficient to justify separating the two specimens as distinct taxa, especially in the absence of additional material. Consequently, we treat the Hainan specimen as conspecific with *H. flavus* (Ashmead, 1905).

## Supplementary Material

XML Treatment for Hemiglyptinus

XML Treatment for Hemiglyptinus flavus
